# Poster 1012: Safety and efficacy of omalizumab in 10 children with asthma and other allergic comorbidities

**DOI:** 10.1186/1939-4551-7-S1-P7

**Published:** 2014-02-03

**Authors:** Rita Aguiar, Pedro Silva, Fátima Duarte, Ana Mendes, Ana Célia Costa, Manuel Pereira Barbosa

**Affiliations:** 1 Immunoallergology Department, Hospital de Santa Maria, CHLN, Lisbon, Portugal

## Background

Omalizumab is a monoclonal anti-IgE antibody approved for the treatment of severe allergic asthma in patients (pts) older than 6 years in Europe. Its efficacy in other IgE-mediated diseases has been the target of several recent studies.

## Objective

Evaluate the safety and efficacy of omalizumab use in allergic pts aged 6 to 18 years, followed in our Immunoallergology Department.

## Methods

We retrospectively analyzed the clinical files of all pediatric pts treated with omalizumab from December 2009 to July 2013. The evaluated parameters included: adverse reactions to omalizumab, clinical evolution, Asthma Control Test (ACT) and Severity Scoring of Atopic Dermatitis (SCORAD) score evolution and medication decrease. Statistical significance was defined by a p value in the appropriate nonparametric test lower than 0.05.

## Results

Ten pts (8M, 2F; mean age 12.9 [7-17] years) were proposed for treatment with omalizumab due to severe uncontrolled allergic disease: all pts had severe allergic asthma, in 3 cases uncontrolled without oral corticosteroids (OCS). Furthermore, 5 children had severe eczema (SCORAD>40), 1 had severe allergic conjunctivitis (AC), 1 was allergic to cow’s milk and experienced multiple anaphylactic events in the course of milk specific oral tolerance induction (SOTI).This pediatric cohort has been treated with omalizumab for an average of 12 [1-42] months. No adverse reactions were attributable to its administration. Average pretreatment ACT scores were 17 [15-19] and improved significantly (p<0.01) after 16 weeks of treatment to 23 [20-24]. In eczema pts, SCORAD also improved significantly (p<0.01) after 16 weeks from 62 [34-90] to 24 [13-35]. All children discontinued OCS: After 2 months in the asthmatics and 3 months in the child with AC. One child with eczema discontinued cyclosporine and one child reduced his daily dose of azathioprine after 1 month. Due to controlled disease, 2 pts were able to initiate specific immunotherapy. A successful cow’s milk SOTI (final daily maintenance dose of 120ml) was possible after 3 administrations of omalizumab.

## Conclusions

In this pediatric cohort, omalizumab was safe and effective not only in the control of asthma but also of other uncontrolled allergic diseases. Furthermore, it allowed for disease-altering immunotherapies to be initiated. Additional studies are warranted for the use of omalizumab in non-asthmatic allergic children, however its role seems promising.

